# Foreign

**Published:** 1840-11-07

**Authors:** 


### FOREIGN.

*On Secondary Depots of Matter. How are Purulent Depositions of Matter formed after Injuries and Operations in parts remote from the original seat of Injury? Illustrated by numerous Cases,* &c. By JOHN CHARLES HALL, M. D. F. L. S.

"Utendum est ætate; cito pede præterit ætas."

OVID.

William T——n, ætat. 35, a man of very intemperate habits, was placed under the care of a surgeon, having, four hours previously, received a kick from a horse on the posterior part of the head. There was a wound, two inches in length, which separated the scalp from the cranium, the bone being denuded of its periosteum to the extent of a crown piece. There was no appearance of fracture or depression. He stated that he was stunned by the blow for some time, but had not been sick. When first seen he was stupid and sleepy; pulse one hundred, strong and full.

Eighteen ounces of blood were taken away from the arm, and a purgative, composed of calomel and colocynth, administered.

6th, (noon.)—Has passed a tolerably good night; not at all drowsy. Complains of a little pain in the head; bowels have acted freely; tongue slightly furred, but moist; pulse hard, and eighty-four; countenance flushed and anxious; skin hard and dry.

9, P. M.—Pain in the head increased. There is a slight puffiness about the edges of the wound; pulse eighty-six, hard and wiry; bled again to ten ounces.

7th.—The blood extracted yesterday was highly inflamed. Has passed a quiet night; no pain in the head; still drowsy and inclined to sleep; pulse eighty-four, more quiet; tongue moist; *the adhesions were broken up, and a considerable quantity of serum escaped.* Apply a poultice to the wound.

15th.—No particular change since our last report. Is now going on well; wound nearly healed. Wants to go home; and says there is nothing the matter with him.

16th.—No change.

17th.—Pain in the head to-day, relieved by purgatives.

23d.—Appears to be going on well; the bone, however, is denuded and rough.

26th.—Up to this day he remained quite well, and was allowed to walk about his room. Violent pain in the head now came on; singing in the ears; with repeated rigors alternating with the most violent perspirations.

10, A. M.—Severe pain in the head; face much flushed; skin hot; tongue loaded; pulse one hundred and twenty, full and sharp. Bled to twelve ounces.

3, P. M.—Pain in the head better; pulse ninety; blood taken away in the morning buffed. An incision was made down to the bone, which was exposed by the accident; it felt rough, but the periosteum was spreading over it; a saline mixture was ordered. In the evening he had another rigor; pulse one hundred and twenty, strong but compressible; blood buffed and cupped; a blister to the head. The medicine to be omitted.

℞. Hydrarg. Chloridi, gr. ii. quâque 4ta. horâ sumend.

27th.—Has passed a very restless night. Had two rigors; one at evening, and the other in the morning: pain in the head; pulse one hundred and thirty-two; cough; no râle, no tenderness of the abdomen; tongue white and loaded; skin hot and dry; slight delirium.

2 o'clock, P. M.—The trephine was applied over the original seat of injury to seek for pus, but none was found; the dura mater was perfectly sound, and not one drop of matter escaped; the wound was closed by suture. Vespere, pulse seventy-two, quiet; no pain in the head, or, to use the poor fellow's own words, "none worth speaking of."

28th.—Has been rather restless during the night. Had a rigor at midnight, another at twelve to-day; slight pain in the head; great intolerance of light and sound: singing in the ears; wound looks rather better; pulse one hundred and twenty, strong and sharp; tongue moist but loaded, particularly at the back part. Bled to eight ounces. Two grains of calomel every eight hours.

29th.—Blood buffed; pain in the head said to be relieved (?) by the bleeding: two rigors at the same time as yesterday: the wound does not look so well to-day; he is less irritable; pulse one hundred and twenty. Continue the calomel.

30th.—Has had a little sleep at intervals during the night; two more rigors; great sickness; pain, with some fulness, in the right hypochondriac and epigastric region, which is increased by pressure; breathing hurried; the wound appears worse; no pain in the head, and no cough; pulse one hundred and twenty; fæces black, fœtid, and particularly offensive; countenance sallow. Omit the calomel; a blister to the epigastrium. Small doses of creosote, to allay the vomiting; and a few grains of sesqui-carbonate of ammonia, with spir. æth. nit. and mint-water, every four hours.

1st.—Two more rigors; no sleep; pain in the loins; pulse one hundred and eight; urine scanty, and high coloured; tongue loaded; skin hot and dry; complains of pain between the shoulders. Beef-tea; two grains of calomel at bed time.

2d.—Has passed a very restless night; had another rigor this morning; breathing occasionally difficult; evidently becoming weaker; the skin, also, is becoming daily more and more yellow; wound unhealthy and languid; pulse one hundred and sixteen.

7th.—The changes that daily took place up to this morning, when he died, require no particular notice; he became weaker and weaker, the symptoms of jaundice gradually increasing.

*Post-mortem examination.*—Head. Upon removing the bone, the dura mater was found to be perfectly free from disease; the longitudinal sinus, over which the trephine had been applied, was healthy, as were all the other sinuses, evincing no trace of previous inflammation. The brain was also perfectly free from disease, nor was there any effusion into the ventricles.

The lungs were congested, and of a dark red colour, with one or two depôts upon each lobe about the size of peas. The liver was much enlarged, the right lobe reaching down below the crest of the ilium. There was a large purulent deposit at the anterior margin, extending for some distance along the inferior concave surface of this lobe; there were also two or three smaller ones on the superior aspect. On cutting through the surface of this lobe, other deposits of different sizes were discovered, being hollow in the centre, and filled with pus; there was also a very considerable abscess in the left lobe. The right shoulder-joint was also affected, and contained a considerable quantity of pus.

We cannot carefully read over this very important and very interesting case without remarking, that, after a comparatively slight injury of the head, a distant organ became secondarily affected, abscesses forming in the liver. A more minute examination will also point out how clearly the condition of the wound proclaimed the disordered condition of the system. Cases of this kind are far from uncommon; and, speaking of them, Mr. Abernethy remarks, "that these circumstances appear to me to be stated rather as occasional, than as occurrences which are common, and naturally to be looked for and expected; and I therefore think myself warranted in supposing that they have not made a sufficient impression on the minds of surgeons in this country at least." Dr. Cheston, in his valuable pathological observations, has recorded several cases of this nature, and we may also add that they have been familiar to surgeons for many years. In the works of Galen we find passages proving him to have been accequainted with them. Bertrandi (*Mémoires de l'Académie de Chirurgie*) mentions several instances in which secondary depôts took place in the liver after injuries of the head.

The subject of secondary depositions of matter taking place, as in the case now before us, in organs far distant from the original seat of the injury, involved, as it is, in mystery, is nevertheless, one of considerable importance, and well worthy of our most careful and attentive consideration. That this is far from being uncommon, after injuries and operations, is a fact that cannot be doubted for a moment. A man is brought into the hands of the surgion, having recieved a blow upon the back part of his head; he recovers from the injury; all pain in the head is gone; he walks about the room, and expresses a wish to return home; suddenly he is seized with pain in the head, succeeded by rigors; there is pain in the right hypochondriac region; the countenance becomes yellow, the symptoms of jaundice daily increase, the man dies, and the lungs, liver, and one or two joints, are found to contain depôts of purulent matter. The question naturally arising is, how came they there? In the greater number of cases they succeed to injuries and operations, frequently after a blow upon the head. Now as they appear, in a majority of instances after accidents, in men who, before the infliction of the injury, were in a perfect state of health, we cannot for a moment suppose that they existed previous to the accident. Such a supposition is not consonant with reason. We cannot suppose such an extensive disease to have existed without disordering the health—without proclaiming its presence by a series of the most alarming symptoms. The disease, therefore, must be created after the accident; the fracture of the bone, or the wound of the scalp covering it, or the inflammatory action produced in one, or both, must have contributed to its production; in what way, however, must form the ground-work of our present investigation.

We define suppuration to be a peculiar process, by which a fluid called pus is formed in the substance or from the surface of parts of the body. The texture in which suppuration seems to be most readily produced by a certain degree of inflammatory action is mucous membrane, whether this lines excretory ducts or canals, or covers the inner surfaces of the respiratory or urinary organs.

Professor Carswell makes a distinction between the process of suppuration, considered as a vital act, and the mere presence of pus as a product of that process. "For if," says he, "pus be found in an organ in which neither the physical nor physiological characters of inflammation are to be detected, either during life or after death, the necessity then of establishing a distinction between the mere presence of pus and suppuration must be obvious." Great violence done to parts is one of the principal exciting causes of suppuration; but simple violence does not always occasion it; "for," remarks John Hunter, "the violence must be followed by something that prevents the cure in a more simple way—something that prevents the restoration of the structure, and the continuance of the animal functions of the part. The parts must be kept long enough in that state into which they were put by the violence, or, what is somewhat similar to this, the violence must be attended with death in a part, as in many bruises, all mortifications, and all sloughs in consequence of the application of caustic which, when the dead parts separate, leave internal surfaces exposed. "And," continues Professor Carswell, in his Illustrations of the elementary Forms of Disease, "formed by a process similar to that of secretion, the chemical composition of pus must vary, not only from the nature of the tissue from which it is derived, but likewise under the influence of various morbid conditions which are known to modify the products of secretions in general." "True pus," says Sir A. Cooper, "has certain properties which, when taken singly, may belong to all other secretions, but which conjointly form the true characteristics of this fluid, viz., globules swimming in a fluid which is coagulable by a solution of the hydrochlorate of ammonia. Pus also contains a very considerable portion of fibrine: thus we find fibrine, serum, and globules, entering into its formation. If I were to hazard a theory upon this subject, I should say that pus was composed of the constituent parts of the blood, slightly changed in their nature by inflammation." (Lectures on Surgery, page 121.)

In addition to what we have already stated, we learn also that the secretion of pus is frequently suspended by disease: thus in fevers the peculiar state of the constitution induced by them has such an effect upon the local affection that a sore will appear to be almost dried up, at any rate its discharge will be very considerably lessened, but as soon as the febrile symptoms subside, pus is again secreted in as large a quantity as ever. The nature of the secretion is also altered by disease, and the appearance of the wound will announce the commencement of constitutional irritation, even before any other symptom is present: the production of true pus ceases when disease attacks either the constitution or the sore; it changes its character, and becomes offensive, thin, and more transparent, containing a greater proportion of the extraneous particles of the blood.

The nature of the fluid most unquestionably varies; varies not only in its nature, but also in its effects. I know of no chemical difference between the pus upon the surface of a common ulcer and that of small-pox; that there is a difference, I presume no one will take upon himself to doubt; but we only are aware of such difference by the nature of the effects produced in each particular form in which suppuration takes place upon the constitution. Doubtless, whatever tends to produce an alteration in the general state of the system influences more or less the nature of this fluid; at any rate renders the human body more susceptible of its influence—more liable to be acted upon by it. If, then, we can discover, by chemical analysis, no essential difference in the constituent parts of this fluid, I am inclined to think that peculiarities in the constitution, or in the nature of the parts in which it is secreted, will account for the varieties we observe in the nature of this fluid. For my own part I have again and again examined gonorrhœal discharges with a microscope, and at the same time pus effused from an ulcer, without being able to detect any difference. Long ago, however, Mr. Howship informed us, that "he could not perceive any essential difference between such discharge and the pus collected from an ulcer." Now every surgeon is well aware that irritation, chemical or mechanical, on the surfaces of the female pudenda, or urethra of the male, which are the seats of gonorrhœa, may excite it. Contusion and forcible distension of the female pudenda will also bring on a discharge, When female children of tender years have been violated, we have always more or less discharge; the introduction of a bougie; the injection of any irritating fluid; connection with women during the period of the menses, or who have a leucorrhœal discharge, will produce more or less inflammation of the urethra, and an acrid secretion that any surgeon would at once pronounce to be gonorrhœa—which is, in fact, a disease of a highly inflammatory character, ending in the secretion and discharge of muco-purulent matter, varying, of course, in different constitutions, and influenced more or less by the general state of the system; capable of being produced in a variety of ways, always, however, commencing with an attack of inflammation, and ending in the discharge of pus—of matter which no one can distinguish from that which is secreted from an ulcer. There is nothing, then, in the nature of this fluid to induce us to suppose that the disease is specific, or that it is even a form of the venereal disease.

We ventured to suggest, at the commencement of this paper, that the proof of the enjoyment of good health before the reception of a blow upon the head, was presumptive evidence of the previous non-existence of the purulent deposits found in the liver after such injuries. In the valuable lectures (now publishing in this journal) of Mr. Phillips, on Surgery, this subject is discussed with considerable ability. "It is difficult," says he, "to explain visceral abscesses as a consequence of surgical operations, though operations are often performed on persons whose general health is good, and in whom we cannot admit that visceral lesions, so grave as those we meet with, can have existed before the injury or operation. Still, as on the one hand observation shows that a great many organic lesions may exist in a latent state, and as, on the other hand, visceral abscesses, consequences of wounds, present, by their multiplicity, their seat, and other circumstances, a great analogy with suppurating tubercles, many persons have maintained that these abscesses were no other than the result of the development of pre-existing tubercles. If this theory ought not to be adopted as a general rule, neither should it be repulsed in all cases. Our opinion is that it should not be admitted as a general rule; unquestionably, in most cases, around these abscesses, phlegmonous inflammation may be detected without tubercles, or tubercular infiltration."

This opinion is doubtless entitled to great respect, admitting, however, of much that can be urged against as well as in favour of its adoption. It appears clear, that to the production of such secondary depôts two things are necessary: first, some exciting cause, as a wound of the scalp or an injury of the cranium; second, a peculiar state of the constitution, either existing previous to the receipt of the injury or produced by some change which takes place in the part itself; for, doubtless, the general state of the system contributes in no inconsiderable degree to the production of certain specific diseases. Thus, a child may escape at one period of the year from rubeola, or any other disease to which children are liable, but at another time, from the strength being exhausted, from a cachetic state of the body, no resistance can be offered by the constitution, and the disease comes on.

Admitting, then, on the one hand, the necessity of an immediate local or exciting cause, which we discover in the wound or blow upon the head, we direct our attention to that state of the system that predisposes to it; to that peculiar condition of the circumstances by which the patient is surrounded: it appears clear, we think, that certain conditions of the atmosphere, or of particular districts—in short, those which contribute to give rise to those fearful maladies, hospital gangrene and typhus fever—tend to produce inflammation of the veins; for I have never seen a case of phlebitis in which typhoid symptoms were not present. We will now enter more fully into the examination of phlebitis, and consider in review what has been written on the subject of inflammation of the veins, and the origin of depôts of purulent matter in certain viscera, at the same time offering such remarks as the cases we have seen, and the post-mortem examinations we have attended, suggest. We will therefore examine, in the first place, the causes of phlebitis; secondly, the symptoms that are present; and lastly, the manner in which the visceral abscesses before alluded to are supposed to be formed, after injuries and operations.

1st. The causes of phlebitis.

In the Cyclopædia of Practical Medicine there is a very interesting paper by Dr. Robert Lee, F. R. S., on diseases of the veins. He there states that he was informed by Sir A. Cooper that he once met with a tumour upon the saphena major vein. This tumour was laid open or removed, and inflammation of the vein soon succeeded, and destroyed life. A lady having a varicose enlargement of the vena saphena, Sir Astley cut it out, compressing the vein below, and requested her to keep quiet. Three or four days afterwards she was labouring under high constitutional irritation, the leg having an erysipelatous appearance; the great saphena vein became inflamed as high as the groin, and the patient died. Mr. Oldknow relates the case of a man who died after the application of a ligature to a varicose saphena vein. A woman was operated upon by Sir E. Home for femoral aneurism; by accident the vein was wounded, and the woman died. "Mr. Abernethy proposed to cut the saphena vein in cases of varicose veins of the leg; proposed, I say, for I believe he never did it; but," continued the learned lecturer, "when I was assistant-surgeon to this hospital, I cut this vein in a poor fellow's leg, and he died of venous inflammation; but still the operation had been performed before with perfect safety." (Notes of Sir B. C. Brodie's Clinical Lectures.)

Inflammation of the veins appears to arise, therefore, for the most part, from direct injury of them; from small punctures, as in bleeding. Mr. Abernethy believed that moving the arm soon after bleeding, produced the disease. Dr. J. Thompson, of Edinburgh, thinks that the state of the lancet as to sharpness has a considerable share in producing the morbid effects. A bad lancet may contribute, Mr. Abernethy thinks, to produce the disease; yet this is not sufficient to account for the accident, without supposing a peculiar irritability of the constitution to be present, and this opinion is borne out by Sir B. C. Brodie, who remarks, "you bleed three hundred patients, and at length one is attacked with inflammation of the veins, and you are at once accused of having a foul lancet, when perhaps it was new, and used only for the first time." Breschet states that punctured wounds, particularly when the instrument is charged with some putrid or irritating matter, are often followed by inflammation of the deep-seated veins, and he attributes the greater frequency of inflammation of the veins of the arm in the present day, to the fact of bleeding with the lancet used for vaccination. We may also mention wounds received in dissection as an exciting cause of inflammation of the veins, and it is reported that Dr. Serrin died from the prick of a pin, which had been used for dressing a blister, and which wound, slight as it was, induced fatal inflammation of the vein. We must also take into consideration the state of our patient at the time he is bled. We must remember that there is more or less of excitement; generally more or less a tendency in the animal body to take on an inflammatory action at the time the operation is performed.

2d. The symptoms of phlebitis.

We are indebted to Mr. Arnott for some very clear explanations of the phenomena attendant upon this disease. The symptoms, he says, manifest themselves in from two to twelve days: "great restlessness and anxiety, depression of spirits, and prostration of strength; sense of weight at the præcordia; frequent sighing, or rather moaning. The common symptoms of fever are present; the pulse is rapid, reaching one hundred and thirty to one hundred and forty in a minute, but is in other respects extremely variable. Under symptoms of increasing debility, and at a time when the local symptoms appear to be subsiding, secondary inflammations of a violent character, and quickly terminating in effusions of pus or lymph, take place in situations remote from the original injury; the cellular substance, the joints, and the eye, have been affected. Death is always preceded by symptoms of extreme exhaustion, such as those of a rapid feeble pulse; dry, brown, or black tongue; teeth and lips covered with sordes; haggard countenance; low delirium."

M. Cruveilhier, whose writings we shall directly more particularly examine, informs us that phlebitis of the bones is one of the most frequent causes of abscesses found in the liver and other viscera. In 1814, he examined the medullary membrane of the long bones of such as had died in the Hôtel-Dieu with abscesses of the viscera, and low typhoid symptoms. There was suppuration in by far the greater number of those of the medullary membrane, sometimes extending throughout the whole length of the bone. Operations upon the bones are extremely likely to produce inflammation of the veins, according to this author; and he refers the constitutional disturbance to a miasmatic state of the system, the whole mass of fluids being injected: and he continues, "however extensive the phlebitis may be, if the pus does not enter the circulation, no accident follows from it; but as soon as the impediment formed by the coagula is removed, atonic adynamic fever, preceded by intense shivering, takes place, and is speedily followed by death."

It appears, then, that the veins are liable to all the morbid changes which are common to the soft parts in general, and that the membrane by which they are lined is peculiarly susceptible of inflammation. This inflammation may be general or local; it may be confined to the vein where the injury was first received; it may spread along the lining membrane to the principal venous trunk; and, in some instances, to the membrane lining the cavity of the heart. Sometimes this inflammation ends in the pouring out of coagulating lymph, by which the vein becomes obliterated; becomes a mere knotty cord. This frequently occurs in horses; in fact, the first case of phlebitis I ever saw was in the jugular vein of a horse of my own, which ended in the loss of the vessel on that part of the neck. When this inflammation is not very acute, it differs little from attacks of a similar nature in any other part of the body. "When," says Mr. Cooper, of University College Hospital, "the secretion of pus is in consequence of inflammation of the membranes lining a vein, the pus is either mixed with the circulating blood, or the inflammation having produced adhesion of the sides of the vessel, at certain intervals boundaries are formed to the collections of the pus, which in this manner form a chain of abscesses in the course of the vessel."

The appearances, then, will vary in our examinations after death, depending as they do upon the length of time the disease has existed. 1st, It may destroy life in five or six days, before pus has formed, although in many cases it is thrown out much sooner. The inner covering then is red and vascular. 2dly, We may have the vein full of coagulated lymph. 3dly, Pus may have been formed in considerable quantities. Now all these effects may be local or general; may be confined to the particular vein injured, or extend along the venous trunks. 4th, We find depôts of matter in the lungs, liver, joints, in parts far distant from the original seat of injury.

3d. How such secondary depôts are formed? We have endeavoured to divide inflammation of the veins into two stages; to prove that the formation of adherent coagulum is the first, and of pus the second period of this disease. The investigations of surgeons also prove to us that the conversion of the first into the second stage of the malady, is frequently produced by irritating parts in a state of active inflammation, by endeavouring at short intervals to extract dead bone, balls, or other foreign bodies; by cramming up an inflamed wound, as in the operation for fistula *in ano*, after the fashion of a portion of the French school; the frequent examination of wounds; the breaking down of newly formed adhesions, by the introduction of a probe or finger. I have twice seen fatal results brought on by this meddlesome surgery, by a system worthy the strongest censure. In his Surgical Dictionary, Mr. Cooper has at great length examined the subject we are endeavouring to discuss; and he reminds us of the important fact that the first effect of every phlebitis is the coagulation of the blood, which becomes adherent to the inner coat of the vessel; such coagulations take place both in spontaneous and traumatic inflammation of the veins. In consequence of the interruption to the current of the blood in the inflamed vein, it becomes stagnant, and effusion of serum takes place in the surrounding parts, unless the other veins are capable of carrying on the circulation. With respect to the local changes attending the suppuration of the veins, the first is the deposition of pus, and M. Cruveilhier observes that this takes place "not between the vein and the clot, but in the very centre of the latter. At first it has the appearance of wine lees, and then it becomes white and opaque. This presence of pus in the centre of clots of blood has led to the idea that these clots are directly organized, and capable both of inflammation and suppuration, in the same manner as it is admitted that the pus or serum, which in pleuritic effusion is circumscribed on every side by a recently formed false membrane, is the product of an exhalation from this membrane itself." But it seems, contends Mr. Cooper, more rational to suppose that the coagulum in phlebitis, and the false membrane in pleurisies, serve, in some measure, as filters, through which the products pass, which are secreted by the internal membrane of the vein, or by the pleura. The presence of pus, then, in the centre of a coagulum, would appear, according to my view, to be a phenomenon of the capillary system.

*Case* II.—W. ——, æt. 23, was admitted for a simple fracture of the femur, and a slight wound of the scalp. The wound in the scalp became puffy in several places, into which free incisions were made. Typhoid symptoms came on; the skin became yellow, as in the last case, and the man died. Upon an examination of the body the two ends of the broken bones were found bathed in pus; there was also an abscess in the liver.

*Case* III.—For some cause or other, (which I do not at this moment remember,) a surgeon applied caustic to the scalp of a gentleman who was his patient, which formed a very large slough. Suddenly he was attacked with a set of very odd symptoms; the abdomen becoming very much enlarged, and he died. Sir B. Brodie opened the body, and found the intestines glued together with lymph. The bone of the head, over which the slough had been made, was inflamed and highly vascular, and the dura mater separated from the bone.

*Case* IV.—A girl was admitted with an extensive wound of the pericranium, by which the bone was denuded. Typhoid symptoms came on, and the girl died. The local appearances were similar to those observed in the last case; the bone being vascular, and the dura mater detached: there was also a very large abscess in the liver.

*Case* V.—A young woman died after a very severe injury of the head. The dura mater was here also found detached, and a large abscess encircling a simple fracture of the thigh.

We have now fully traced the effects produced by those injuries—have proved such injuries to be the exciting causes of the various purulent depôts discovered in parts far distant. But we call in vain to our assistance the aid of anatomy to draw aside the veil that conceals these phenomena. The peculiar construction of our textures, which our forefathers concluded allowed the fluids to wander from one part of the body to the other, as through a sponge—in one word, the whole system of organism—is incapable of accounting for so extraordinary a circumstance.

An inflammation which seemed to have nothing to do with that now under consideration (phlebitis) has filled up the great void that seemed to separate the suppurating wound from a visceral abscess: a series of experiments appear to have clearly established this proposition,—that every foreign body introduced into the veins in the living subject occasions, when its discharge by the emunctories is impossible, visceral abscesses completely resembling those consequent to wounds and surgical operations, and such abscesses are the result of capillary phlebitis in these same viscera. (Translated from *Nouv. Bibl. Med.*)

The experiments of Cruveilhier are certainly very clever, and tend to throw some light upon the subject under examination. That we may rely upon them I have no doubt; for having taken the trouble to make some of them myself, I found what this gentleman has stated to be perfectly correct. I did not certainly try all of them, and for two reasons; first, because I had not sufficient time to devote to them; and secondly, I wished not, for the purpose of idle curiosity, to put numerous dogs to the most fearful torture. When any practical end can be gained by such means, we have an undoubted right to seize upon every source whence information can be drawn; but the truth of such experiments having been confirmed, it is wanton cruelty to torture poor brutes for no good purpose, nor can such deeds be too strenuously condemned.

We shall now conclude this part of our examinations, by recording a few of the experiments to which we have alluded, and making such comments as they seem to demand. It is a question of some interest whether suppuration ever takes place without the existence of previous inflammation, and Cruveilhier contends that this can never be the case; but collections which he describes as extraneous matter may form in various parts of the body without such parts having been attacked by inflammation. Dr. Thompson doubts, however, "whether these collections of matter ever form without inflammation, and is inclined to believe that in whatever texture or part of the body scrofula manifests itself, these inflammations will be found to exist. The phenomena of inflammation, both local and general, are, it is true, modified by the existence of the scrofulous diathesis; but they are, I believe, always present in such a degree as to justify us in giving to them the name of inflammation, and in classing most, if not all local scrofulous affections, among inflammatory diseases."—*Thompson on Inflammation.*

John Hunter observed, that pus does not irritate the peculiar surface by which it is produced, although it may be highly exciting to any other, and, therefore, that no suppurating surface of any specific kind can be kept up by its own discharge, for if this were the case, any sore secreting an acrid and irritable fluid would be kept open by its discharge, and would never be induced to heal; and this may also be said of many other fluids: thus the bile, the urine, and the tears, do not excite the particular parts, the glands or ducts by which they are secreted, and yet they are nevertheless capable of irritating any other part of the human frame. From this I think, then, that we may very justly draw the conclusion that when pus once enters a vein, once mingles with the circulating blood, whatever may have been its previous nature, whatever effect it may have produced upon the part where it was created, it now either has changed its condition, or, acting as a foreign body, becomes highly irritating to the particular parts to which it is applied.

"If," says M. Cruveilhier, "any irritating fluid is thrown into the femoral vein of a dog in the direction of the heart, (which can be accomplished after a few of the valves are broken down,) and the collateral veins do not convey the liquid into the circulation, the injection proves immediately fatal; the limb in thirty-six hours becomes swollen; and if the animal then dies, or is killed immediately, bloody extravasations are found in the substance of the muscles, and in the cellular tissue of the limb. The large veins are distended with adherent and coagulated lymph or blood, and the small veins corresponding to the extravasations are also full of concrete blood, while those appertaining to the healthy parts are free. If the animal survives the experiment, collections of pus replace those of blood, at the same time that pus is substituted for the coagulated blood in the veins."

This physiologist next devoted his attention to endeavour to find out what became of the pus in local inflammation of the veins, when such fluids became mingled with the blood. When, however, it was so blended, it was difficult to discover its presence, and therefore quicksilver was employed.

"If a large quantity of quicksilver be injected into the femoral or jugular vein, the animal will become exceedingly depressed, and perish in from twelve to twenty-four hours, in a state very analogous to that observed in chronic catarrh. The whole of the mercury will be found again in the lungs, which will not be inflamed, but gorged with serosity that may be pressed out of them. But if the quantity of quicksilver be smaller, the animal will survive the experiment for a longer period, and then there will be perceived an induration around each globule of the mercury; in a later stage collections of purulent matter may be discovered."

A variety of these experiments M. Cruveilhier offers for our examination, varied as they have been by him a thousand different ways, and always with the same result.

The liver being the seat of a particular system of veins, which veins are destitute of valves, and have numerous windings in the mesentery, he says, "I next drew out a knuckle of intestine, and injected quicksilver into one of the mesenteric veins. In a dog which survived this operation twenty-four hours, the liver was studded with red, superficial, and slightly prominent patches, of the colour of wine-lees, and its texture, when cut into on a level with these patches, presented the same colour to the depth of four or five lines. In the centre of each small red induration was a globule of quicksilver, a certain quantity of which had penetrated into the small veins. In another experiment on a dog which had an umbilical epiplocele, quicksilver was injected into a small vein of the omentum. In about ten weeks the animal was destroyed. The liver was studded with numerous yellowish tubercles, some of which lay near its surface, others in its substance, and each having in its centre one or more globules of quicksilver. Some of them presented two distinct strata; one of a tubercular substance at the circumference, the other of puriform matter in the centre, in the middle of which were the mercurial globules. These observations seem clearly to prove that all extraneous bodies introduced into the general circulation are inevitably conveyed to the lungs, and such as enter the abdominal venous circulation as certainly proceed to the liver; these viscera constituting a barrier, which they pass not beyond, except in certain cases. These experiments, observes Mr. Cooper, solve one difficulty, which clinical observations alone could never have solved: "How, in the hypothesis concerning phlebitis, is the pus conveyed from the general venous system into the capillary system of the liver? Should not the pus stop in the capillary vessels of the lungs? It seems as if abscesses should only take place in the latter organs; yet experience proves that abscesses of the liver are very common after injuries and surgical operations, notwithstanding that the capillary system of the liver only communicates directly with the vena porta and the hepatic veins; but this objection is at once reduced to its proper value by the demonstration of that subtle liquid, quicksilver, passing completely through the general and capillary system of the liver when injected into the branches of the vena porta, and, in other cases, passing through the general and pulmonary capillary systems, or, what is still more convincing, pervading several times the different orders of capillary vessels."

Professor Cruveilhier considers it, therefore, to be clearly made out that the pus which is introduced into the blood is retained in some part or other of the capillary system; that its tendency is to excite every where capillary phlebitis; that this inflammation is more likely to take place in the lungs than any other part; next in the liver, and next in the spleen; in fact, it appears clearly proved that pus, like quicksilver, once taken into the circulation, may be detained in the lungs or liver, or any other part of the system; it then produces circumscribed spots of inflammation, and this proceeds more or less rapidly to a state of suppuration.

This certainly appears a much more rational way of accounting for the secondary depôts found in various parts of the body, after injuries of the head, and various surgical operations, than to suppose that they existed previously in the parts where they are discovered, and were excited to take on a new sphere of action by some peculiar state of the system induced by the wound of the scalp, or the injury done to the bones.

We have been led already to discuss this subject at greater length than we at first intended. There is, however, another query which must, if possible, be answered, viz., why do not visceral abscesses take place in cases of copious accumulations of matter, as in chronic pleurisy and peritonitis? and, secondly, is a wound necessary to their development? Quesnay has noticed a great difference in relation to consecutive effects between abscesses of long standing and the suppuration from recent wounds. Now to what are we to trace this difference? Are we to conclude that absorption of pus takes place in the one and not in the other? We know that a large abscess, the opening of which has been delayed, sometimes disappears, its fluid contents having been absorbed and taken into the system; yet it appears that the constitution does not suffer, and that this extraneous matter is thrown off by the various outlets of the body; therefore it would certainly appear that there is a very considerable difference between the effects produced by the introduction of this fluid at once into the circulation, and the introduction of similar matter by previous absorption.

M. Cruveilhier, in conclusion, adds, that what M. Dance proposed as a conjecture he has proved to be true,—that in several cases of "wounds of the head the veins of the diploe have been found purulent, and this state co-existing with numerous abscesses of the liver and lungs. Several convincing preparations of this were presented to the Anatomical Society, and at the present time it may be announced as a demonstrated truth, that, in cases of wounds of the head, the visceral abscesses of the liver, the lungs, and the spleen, are the consequences of phlebitis, and more especially phlebitis of the diploe; but the observation that inflammation of the veins of bones is a cause of visceral abscesses applies not only to the veins of the diploe, but to all the veins of bones; and I lay it down, as a general proposition, that phlebitis of the bones is one of the most frequent causes of visceral abscesses after wounds and surgical operations implicating the bones."

This conclusion is at any rate as rational as any we can come to, and far more so than many of the theories that have been from time to time advanced. The subject, however, is overshadowed by clouds and difficulties that the scalpel of the anatomist cannot break through. It is surrounded at present by a veil that human talent and understanding in vain endeavour to penetrate.

But our examination has led to some satisfactory results, inasmuch as we have discovered some of the more frequent causes of phlebitis. We are obliged to extract blood from the arm, but we are not obliged to induce death by passing a thread round a vein, or by breaking down newly-formed adhesions with a silver probe; and, therefore, although unable to trace the disease through all of its ramifications, we nevertheless are able to discover some of the more frequent causes producing it, and, if we avoid them not, we are not only wilfully blind, but culpably negligent also, and answerable for the lives we thus destroy. But this leads us to consider next the treatment of that peculiar state of the system which we have thus considered, and which we have found to arise after injuries and operations.

We have divided inflammation of the veins into two stages: first, adhesive inflammation, and second, a more advanced period comes on, when we find that this stage has passed away, and the suppurative process commenced. This view of our subject will be found of practical importance in our prognosis. I have not, be it remembered, spoken of inflammation of the veins arising without any apparent cause; traumatic phlebitis has more particularly occupied our attention; but in the Cyclopædia of Practical Medicine, Dr. R. Lee has taken up this part of the subject in a manner that leaves me nothing to say; in a manner that will amply repay an attentive perusal, and from his long and patient examination of uterine phlebitis, his remarks are entitled to the respect the opinions of this gentleman always demand. We may, however, be allowed to say that uterine phlebitis is known to be one of the worst forms of puerperal disease, and that, next to inflammation of the veins from wounds, one of the most common cases is phlebitis of the lower limb, consequent to uterine or hypogastric phlebitis; but this may arise under two distinct conditions of the body:

1st. After parturition.

2d. In cases of cancer of the womb.

We have stated the division here made is of practical importance, inasmuch as it is only during the first or adhesive stage, during that very early period of the affection when the blood is just beginning to coagulate within the vessel, that the cure can be attempted with any reasonable hope of success; for when the second stage comes on, when pus is formed and introduced into the system, medicine is of little avail; at least, all the remedial agents with which we are yet acquainted. Our treatment must be both local and general; copious bleeding, both with the lancet and with leeches, cold lotions to the part, and the free administration of calomel and opium. This is all we know at present of the treatment of the disease; but when the second stage comes on, the lancet and leeches can do no good. The quick and feeble pulse, the brown tongue, the cold wet hands, the muttering delirium, point out what it will be proper to administer. Wine, brandy, and then opium and ammonia, will be the best medicines; clysters of strong broth must be thrown up, and every means taken to support the declining powers of life; but they hold out little or no hope of success, and in spite of all we can do our poor patient is hurried to the grave.

There yet remains one point of practical importance in the treatment of phlebitis that must not be overlooked, as it is important to know at what period we are to give up extracting blood. We answer, so soon as the second stage has commenced. True, I may be answered, "by taking away the blood you remove also a portion of the poison." Granted; but in taking away the blood you also lessen the powers of reaction, and the mere taking away of this fluid does not prevent the secretion of pus, which goes on as rapidly as ever.—*Lon. Med. Gaz.*

